# Probable sarcopenia and depressive symptoms in community-dwelling older adults: exploring the role of frailty and comorbidities

**DOI:** 10.1007/s40520-025-03005-8

**Published:** 2025-03-25

**Authors:** Emanuele Rocco Villani, Andrea Salerno, Federico Triolo, Laura Franza, Giulia Vaccari, Barbara Manni, Antonella Rita Vaccina, Lucia Bergamini, Vanda Menon, Davide Zaccherini, Andrea Fabbo

**Affiliations:** 1 Dipartimento dell’Integrazione, UOC Geriatria Territoriale, AUSL Modena, Modena, Italy; 2 Department of Neurobiology, Aging Research Center, Care Sciences and Society, Karolinska Institutet, Stockholm, Sweden; 3 Emergency, Anesthesiological and Reanimation Sciences Department, Fondazione Policlinico Universitario A. Gemelli-IRCCS of Rome, 00168 Rome, Italy; 4 Emergency Department, Azienda Ospedaliero-Universitaria di Modena, Largo del Pozzo, 71, 41125 Modena, Italy; 5 Director of Public Health, AUSL ASTI, Asti, Italy

**Keywords:** Aging, Sarcopenia, Depression, Frailty

## Abstract

**Background:**

Sarcopenia is a syndrome characterized by the loss of skeletal muscle associated with reduced physical strength/performance and could be correlated with depression, that is the most frequent cause of emotional distress in old age and can reduce the quality of life of the older adults.

**Aim:**

The aim of the present study is to evaluate the association between probable sarcopenia and depressive symptoms in older adults, and the impact of comorbidity and frailty on this association.

**Methods:**

This cross-sectional study included community-dwelling older adults at their first geriatric evaluation. Probable sarcopenia was screened according to SARC-F. Clinically significant depressive symptoms (CSDS) were assessed according to the 5-items geriatric depression scale (GDS.) Frailty was determined through the CHSA-clinical frailty scale (CFS). Comorbidity burden was scored through the Cumulative Illness Rating Scale-Geriatric (CIRS-CI).

**Results:**

We included 238 participants with a mean age of 82.4 (± 6.9) years of age, 152 (63.6%) participants were females. Probable sarcopenia was diagnosed in 131 (55.0%) participants, while CSDS were present in 186 (78.2%) participants. In the multiadjusted model, probable sarcopenia was associated with a higher likelihood of CSDS (OR 2.70, 95% CI 1.03–6.12). No significant interaction of frailty and CIRS were found on the association between probable sarcopenia and CSDS.

**Conclusions:**

Sarcopenia and depressive symptomatology are highly co-occurring in geriatric patients, and this association may be independent of frailty and comordibity burden.

## Introduction

Sarcopenia is a multifactorial condition, classified within the ICD-10-CM as M62.84 [1], characterised by a reduction in both muscle mass and strength. The prevalence of sarcopenia ranges from 5 to 50%, depending on a person´s age [2]. Sarcopenia affects more than 50 million individuals worldwide, but this number could exceed 200 million in the next 4 decades [3]. Sarcopenia has several major consequences, both economic and social, and the direct cost of sarcopenia has accounted for 1.5% of total healthcare expenditure in recent years [4] Moreover, it tends to be more prevalent in special populations (i.e. old adults, people with Down syndrome, cancer patients, etc.) [5].

Depression affects up to 13% of the older population and is cause of emotional distress, affecting every aspect of a person’s life, it is related to poor health and suicidal behaviour, and is overall a major problem in the older population [6]. Depression has been linked to body composition, muscle strength, and disability, which are all important for quality of life and well-being [7]. Both depression and sarcopenia are common in older adults and share similarities in aetiology, prognosis and risk factors, e.g., lack of exercise, upregulation of inflammatory factors, and hormonal disorders of the hypothalamic–pituitary–adrenal axis [4]. Thus, it has been observed that patients with sarcopenia are more likely to have depression and experience worse outcomes [8]. But even though several studies have found an association between sarcopenia and depression, results have been inconsistent. The most updated metanalysis conducted by Li et al. included 19 studies for a total number of 16,869 persons, with a mean age of 73 years old. It showed a significant positive association between the two conditions, even though a significant heterogeneity was also observed. The reasons behind the high heterogeneity are mainly due to different diagnostic criteria of sarcopenia and depression [9]. It is also worth noting that other conditions that could act as effect-modifiers on their association have scarcely been evaluated. One geriatric condition that could play an important role in modulating the relation between sarcopenia and depression is frailty. Frailty is a “clinical state characterized by a decrease of an individual's homeostatic reserves, responsible for enhanced vulnerability to endogenous and/or exogenous stressors” and it has been repeatedly associated with several negative health outcomes, including depression [10]. While the physical phenotype of frailty shares some characteristics with sarcopenia, the two conditions are not the same, and one is not necessary to the other, even though they are deeply intertwined [11]. Sarcopenia, depression and frailty are also associated to the presence of comorbidities [12], suggesting the strong underlying relations between all these conditions.

The aim of the present study is to evaluate the association between probable sarcopenia and depressive symptoms and to evaluate the concomitant role of frailty and comorbidity among community-dwelling older adults.

## Methods

### Study design and population

Sarcodepth is a retrospective cross-sectional study. Participants with complete clinical charts were consecutively enrolled among patients attending one of the seven outpatient geriatric clinics located across the province of Modena, Italy (Modena, Mirandola, Carpi, Sassuolo, Castelfranco Emilia, Vignola, Pavullo nel Frignano) between June 2020 and October 2021. Eligible participants were adults older than 65 years, living at home and at their very first geriatric evaluation. The exclusion criteria were life expectancy < 6 months and a Mini Mental State Examination (MMSE) ≤ 10.

### Probable sarcopenia

For the purpose of our study, the Italian version of the SARC-F was administered to screen for probable sarcopenia [13]. SARC-F is a 5-item questionnaire for screening sarcopenia based on limitations in strength, walking ability, rising from a chair, stair climbing and falls. SARC‐F scale scores range from 0 to 10 (i.e. 0–2 points for each component; 0 = best to 10 = worst) and are dichotomized to represent symptomatic (4 +) vs. healthy (0–3) status. Strength is measured by asking respondents how much difficulty they had lifting or carrying 10 lbs (0 = no difficulty, 1 = some, and 2 = a lot or unable to do). “Assistance walking” is assessed by asking participants how much difficulty they had walking across a room and whether they use aids or need help to do this (0 = no difficulty, 1 = some difficulty, and 2 = a lot of difficulty, use aids, or unable to do without personal help). “Rise from a chair” is measured by asking respondents how much difficulty they had transferring from a chair or bed and whether they used aids or needed help to do this (0 = no difficulty, 1 = some difficulty, and 2 = a lot of difficulty, use aids, or unable to do without help). “Climb stairs” is measured by asking respondents how challenging is climbing a flight of 10 steps (0 = no difficulty, 1 = some, and 2 = a lot or unable to do). “Falls” is scored a 2 for patients reporting falls four or more times in the past year, 1 for those reporting falls 1–3 times in the past year, and 0 for those with no falls in the past year. The scores range from 0 to 10 points, with 0 to 2 points for each item, and a score equal to or greater than 4 accounts for probable sarcopenia [13].

### Depressive symptoms

In this study, depressive symptoms were assessed using the Geriatric Depression Scale-5 (GDS-5), a validated screening tool specifically designed to capture depressive symptomatology in older adults, rather than diagnosing major depressive disorder (MDD) according to DSM-5 criteria. For the purpose of our study, the Italian version of the 5-item GDS was administered [14]. A cut-off of 2 has been shown to be both sensible and specific to indicate the presence of clinically significant depressive symptoms (CSDS) [15]. While longer versions of the GDS, such as the 15-item or 30-item versions, may offer slightly higher specificity, studies have shown an acceptable correlation between the GDS-5 and these extended forms [15].

### Covariates

Demographics factors such as age and gender were collected for all participants. Cognitive impairment was screened through the Mini Mental State Examination (MMSE), which consists of 30 items and evaluates orientation, concentration, attention, verbal memory, naming and visuospatial skills. The total score is adjusted according to the recipient’s educational background and age [16]. A score between 26 to 30 indicates no cognitive impairment, a score between 20 to 26 indicates mild cognitive impairment, a score between 10 to 20 indicates moderate cognitive impairment and a score < 10 indicates severe cognitive impairment [17].

Frailty was evaluated according to the Canadian Health Study Clinical Frailty Scale (CHSA-CFS). It is scored on a scale from 1 (very fit) to 9 (terminally ill), in which the patient should be in one category, and is based on clinical judgment. Each point on this scale corresponds with a written description of frailty, complemented by a visual chart to assist with the classification of frailty. A score ≥ 5 is considered to be frail [18]. For the purpose of the study, the cumulative illness rating scale (CIRS) comorbidity index was collected to evaluate comorbidity states. CIRS is a standardized instrument for rating medical problems, classifying them through organs or systems [19].

Information on drug therapy was obtained through clinical charts of the participants at the moment of the first visit, and recorded according to their Anatomical Therapeutic Chemical code (level 4), to identify antidepressant use.

Functional dependence was defined according to the basic Activities of Daily Living (BADL) scale: a score of 6 indicates no dependence, 3–5 indicates mild dependence and 0–2 indicates severe dependence [20].

All the evaluations were conducted by the examining physician.

### Statistical analysis

The baseline study sample characteristics were compared according to the presence of depressive symptoms, and reported as mean and standard deviation, or counts and proportions (%), as appropriate. Analysis of variance and chi-square test were used to compare the distribution of continuous variables and categorical variables, respectively.

The association between depressive symptoms and probable sarcopenia was tested through a multivariable logistic regression model, including interaction terms for CFS and CIRS. Specifically, a two-way interaction term was added to assess whether the modifiers (CFS and CIRS) influenced the effect of the exposure (sarcopenia) on the outcome (depressive symptoms). The combined OR (Mantel–Haenszel test) of the variables with a p > 0.10 at the univariate analysis was evaluated. Variables whose combined OR was 10% different from the crude OR, as well as demographics, were considered as covariates.

R-studio 4.1.2 for Windows was used for all analyses [21]. The packages dplyr [22] and officer [23] were used.

### Statement of ethics

The study was approved by the local ethical committee (AVEN) under the number 88/2023. The study was performed in accordance with the Declaration of Helsinki and all participants gave written informed consent before entering the study.

## Results

**Fig. 1:**
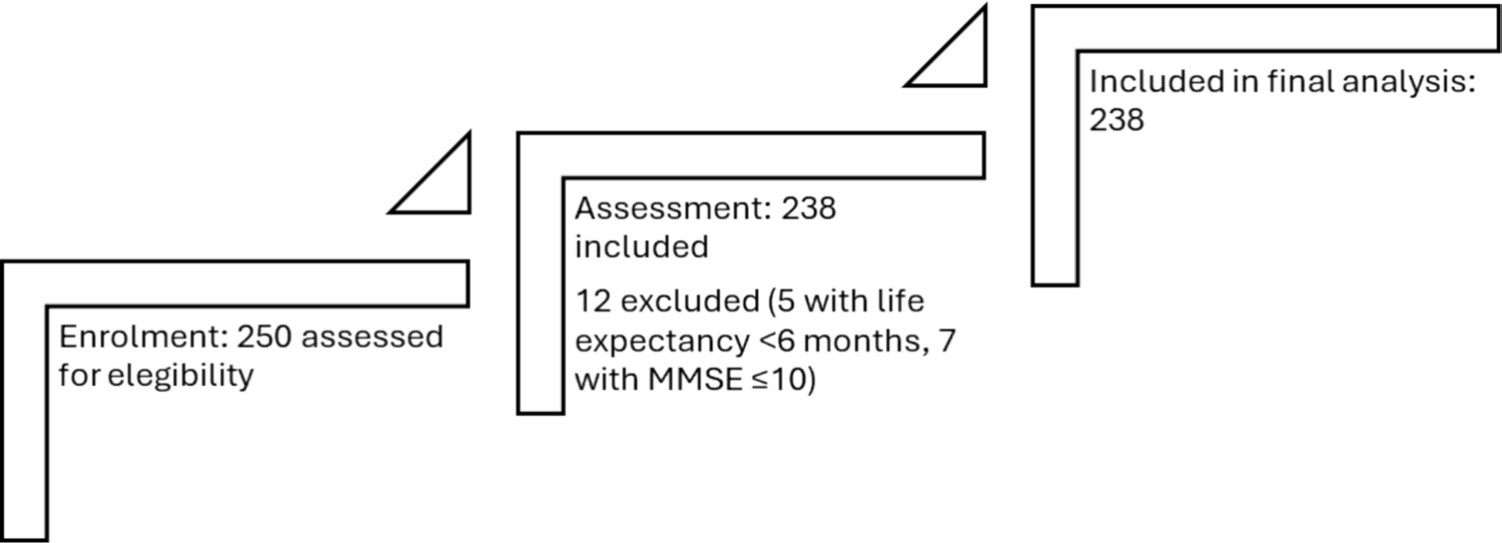
Flow diagram to illustrate participant flow

The analytical sample included 238 participants with a mean age of 82.4 (± 6.9) years of age, 152 (63.6%) participants were females. Probable sarcopenia was diagnosed in 131 (55.0%) participants, while CSDS were present in 186 (78.2%) participants. A flow chart on how participants were included is present in Fig. 1.

The main characteristics of the study sample, subdivided in two groups according to the presence of depressive symptoms, are shown in Table 1. The majority of participants with CSDS were women. Prevalence of sarcopenia was higher among participants with CSDS. Prevalence of moderate dependence in ADLs was higher among participants with CSDS. Frailty was also more prevalent among participants with CSDS. CIRS comorbidity index was similar between the two groups. Regarding cognitive impairment, no differences were found between the two groups. Antidepressants were similarly prescribed between the two groups.

**Table 1:** Sample characteristics

Variable	Overall (n = 238)	5-items GDS < 2 (n = 52, 21.8%)	5-items GDS > = 2 (n = 186, 78.2%)	p-value
Age (mean, SD)	82.4, 6.9	82.4, 7.0	82.2, 7.2	0.863
Female sex (n, %)	152, 63.6%	27, 51.9%	125, 67.2%	**0.043**
Probable sarcopenia (n, %)	131, 55.0%	25, 48.1%	106, 56.9%	** < 0.001**
CIRS Comorbidity Index (mean, SD)	5.9, 1.8	6.3, 2.1	5.9, 1.8	0.122
ADLs (n, %)				**0.002**
0–2	67, 28.1%	24, 46.2%	43, 23.1%	
3–5	102, 43.4%	20, 19.6%	82, 44.1%	
6	69, 28.6%	8, 15.4%	61, 32.8%	
ADLs (mean, SD)	3.8, 1.6	4.7, 1.7	3.8, 1.9	**0.001**
Frailty (n, %)	140, 58.9%	23, 44.2%	107, 62.2%	**0.021**
MMSE (n, %)				
24–30	73, 30.7%	17, 32.7%	56, 30.1%	0.401
20–24	102, 42.3%	25, 48.1%	77, 41.4%	
10–20	63, 26.4%	10, 19.2%	53, 28.5%	
MMSE (mean, SD)	22.8, 6.2	22.4, 5.7	22.9, 6.6	0.723
Antidepressants (n, %)	61, 25.1%	9, 17.3%	52, 28.0%	0.120

Bold indicates significant values

Table 2 shows the results of the logistic regression analysis. While in the univariate analysis female sex, probable sarcopenia, frailty and disability were associated with a higher likelihood of CSDS, in the multivariable analysis only probable sarcopenia was associated with a higher likelihood of CSDS (adjusted OR 2.70, 95%CI 1.03–6.12). No significant interaction on CSDS was found regarding frailty and CIRS on the association between probable sarcopenia and CSDS. Due to the low prevalence of antidepressant use in the study population, adjustment for their use was not considered, as it would have likely led to unstable estimates in the regression model.

**Table 2:** Logistic regression analyses

Variable	OR (95% CI)*	aOR (95% CI)**
Age	1.00 (0.96–1.04)	1.03 (0.98–1.09)
Female sex	**1.90 (1.02–3.54)**	1.71 (0.87–3.33)
Probable sarcopenia	**3.40 (1.74–6.62)**	**2.70 (1.03–6.12)**
Frailty	**2.08 (1.11–3.89)**	1.06 (0.45–2.48)
CIRS comorbidity index	1.11 (0.93–1.33)	1.05 (0.83–1.28)
Antidepressants	1.57 (0.56–14.25)	1.10 (0.66–11.01
ADL disability		
- None	REF	REF
- Moderate	**1.29 (1.14–4.60)**	2.07 (0.95–4.57)
- Severe	**4.26 (1.75–10.37)**	2.21 (0.64–7.99)
MMSE	1.01 (0.97–1.06)	1.05 (0.98–1.11)
Probable sarcopenia:Frailty	NA	7.10 (0.50–13.75)
Probabile sarcopenia:CIRS	NA	1.29 (0.85–3.19)
Frailty:CIRS	NA	1.60 (0.21–1.78)

Bold indicates significant values

*CI* confidence interval, *NA* not assessed. *univariate analysis, **model adjusted for age, sex, CIRS, antidepressants, frailty, disability and the interaction term.

## Discussion

The present study investigated the possible association between depressive symptoms and probable sarcopenia in a population of older outpatients at their first geriatric evaluation and whether this association is impacted by frailty and comorbidity. One issue in clinical practice is that there are several operative definitions of sarcopenia and different methods to evaluate it [6], but a quantification of muscle mass is always needed. According to a recent metanalysis, SARC-F is an effective screening tool for sarcopenia in the elderly, characterized by low to moderate sensitivity and moderate to high specificity, according to the different operative definition of sarcopenia adopted [14] and is highly valuable in diagnosing a probable sarcopenia. A similar issue is also present for depression; in the geriatric population, subclinical depressive symptoms are common and can significantly impact health outcomes, even in the absence of a formal psychiatric diagnosis [24]. Using the GDS-5 score offers the possibility of identifying this subset of the population, which is sometimes overlooked.

A significant association between probable sarcopenia and CSDS was found, and it was not affected by the presence of frailty or comorbidity burden.

We observed that CSDS had a high prevalence in this population (78.2%), which was more than what was expected according to previous studies [25, 26]. We cannot exclude a possible selection bias, because even if the patients enrolled in this study were not specifically referred to evaluation for mood or cognitive issues, our clinic is specialized in following both. Also, sarcopenia was a prevalent condition in the study population (55,0%), even though its prevalence was in line with previous observations of comparable mean age [2].

We found that probable sarcopenia was more prevalent among people with CSDS than in people without CSDS. These findings are in line with the metanalysis by Li et al. [9] and with a perspective study they later conducted: low muscle mass was significantly associated with CSDS. Chen et al. also observed a substantial association between depressive symptoms and gait speed [27] and, in another study, they found that the incidence of new depressive symptoms increased with sarcopenia, and that a higher level of muscle mass was protective against the development of depressive symptoms [28]. Sarcopenia can be targeted in different ways, often through multicomponent strategies incorporating nutritional, exercise, and pharmacological strategies, but the impact of these strategies on depressive symptoms is scarce [29].

In previous studies, the validity and strength of the association between CSDS and sarcopenia in community-dwelling older adults was evaluated considering potential confounders, such as age, sex, BMI, education, leading to unclear associations with gender, and being overweight/obese [8]. To our knowledge, no study has explored the role of variables like frailty and comorbidity: measures of frailty were not evaluated in any of the studies included in the metanalysis by Li et al.[9]; similarly, the authors could not gather data according to comorbidity due to the low number of studies that accounted for it [30, 31].

Concerning frailty, literature shows that depression and frailty are two common conditions among older adults. A systematic review and metanalysis by Soysal et al. showed that approximately 40% of individuals with depression suffer from frailty, and a similar proportion of those with frailty has depression [10]. Nevertheless, in our study frailty resulted higher among participants with CSDS, but such association was not confirmed in the multivariate analysis. This could mean that the link between depression and frailty is association rather than causation, possibly because some items comprised in the screening tests for depressive symptoms and frailty investigate similar aspects, e.g., reduced mobility and weakness, which can be due to both muscular diseases and depressive apathy. Moreover, in our opinion, some patients with frailty may not report complaints or may not be aware of their condition, especially when frailty is severe [32], thus these patients may not show significant depressive symptoms associated to their condition. Although no significant interaction was found between frailty and probable sarcopenia in relation to CSDS, this does not necessarily imply the absence of a true effect. The lack of significance may be due to limited statistical power, residual confounding, or the inherent limitations of the frailty assessment tool used. Additionally, the Clinical Frailty Scale can be affected by operator’s interpretation and may not fully capture the complexity of frailty-related factors that could influence depressive symptoms.

Regarding the relationship between depression and comorbidity, in our study no association has been observed. Nevertheless, literature has provided with a large amount of evidence of the association between these two conditions. Higher prevalence of depression has been found in patients with a range of conditions including cardiovascular diseases (e.g. myocardial infarction) [33], diabetes and arthritis [34]. A greater prevalence of depression has been found in people who had at least one chronic physical condition compared to those with none [35]. A meta-analysis by Chang-Quan et al. found that older adults with a chronic physical condition had a higher risk for depression [36]. To suggest a more than an additive effect, a meta-analysis by Read et al. [37] found that people with multimorbidity had twice the risk of depressive disorder compared to those without multimorbidity, and almost three times the risk compared to those with no chronic physical condition, even though it is worth noting that the studies taken into consideration were cross-sectional, which may have influenced the analysis. Further, people with depressive symptoms may be less likely to adhere to their medical regimens, contributing to a poorer disease management and an increasing risk for multimorbidity [37]. According to what has been reported in literature, it might be surprising that our study did not detect an association between CSDS and comorbidity. The explanation for this phenomenon could lie in the fact that in our study the comorbidity was calculated using the CIRS, a tool that "collapses" into a single numerical value all the pathologies from which the patient suffers, losing specificity on the nature of the pathologies themselves; this is important since, as mentioned above, some diseases are more associated than others with CSDS.

In our sample, use of antidepressant drugs was less prevalent than CSDS: only 25.1% of the participants were taking antidepressant medications, in comparison with 78.2% suffering from CSDS, suggesting a matter of under-treatment of this condition in geriatric outpatients. Also, GDS-5 allows to identify sub-clinical depression, for which there is no consensus on the use of antidepressants [38]. Surprisingly, the proportion of patients using antidepressants was similar between the two examined groups: it is possible that some patients had a remission of depression with the medication, thus being included in the group of patients without CSDS but using antidepressants, while other patients had no benefit from the prescribed drug, remaining in the group of patients with CSDS. It is also worth noting that, on the one hand, antidepressants take time to have an effect, and, on the other hand, they tend to be less effective after chronic treatment, thus some patients may not have had time to benefit from the therapy, while others may no longer feel the beneficial effects [39].

### Limitations

The present study has some limitations. A first limitation concerns the study definition of depressive symptoms, which is somewhat ambiguous. Depressive symptoms may be caused by life events (such as mourning) and many medical conditions. In some studies, focusing on depression and sarcopenia in the elderly population, it was even decided not to evaluate patients who were using antidepressant medications, to avoid possible interferences [40]. Second, GDS is an effective instrument for assessing depressive symptoms in elderly people, but it could result positive also due to physical illnesses that restrict mobility. GDS takes into account only psychological manifestations of depression, not considering other symptoms, like anorexia, insomnia, cognitive dysfunction, which are even more frequent in senile age. Third, sarcopenia, depression and other study variables were evaluated using screening tools and not diagnostic tests and screening tools are calibrated to maximize sensibility, not specificity. Fourth, comorbidity has been evaluated only with the CIRS comorbidity index, and not with the severity index, which could have eventually revealed an influence of comorbidity on the relationship between depression and sarcopenia. Fifth, the study population may have suffered from a form of selection bias, due to the necessity to receive a geriatric evaluation. The involvement of a community-based sample recruited through attendance at a geriatric clinic has excluded frailer and home-bound individuals, which is something that could be addressed in future studies, as it was impossible to evaluate the relation between depression and sarcopenia in this specific subgroup [41]. Finally, the study had a cross-sectional design, so we could not establish a causal or temporal relationship between sarcopenia and depression.

### Strengths

The present study has also many strengths. First, SARC-F, GDS-5, MMSE, CIRS and ADL/IADL are quick and easy tests, with low inter-operator variability, largely validated for a geriatric multidimensional approach, which allows for reproducibility. Second, the introduction of CFS has allowed to evaluate the impact of frailty on the strength of the association. Third, the mean age of our sample was higher than that of participants in other studies, leading our study to be one of the first involving an oldest old population. Fourth, our study was conducted in a European country, while most of the studies included in the metanalysis were conducted in Asia.

## Conclusions and future perspectives

The results showed that the prevalence of probable sarcopenia was high in patients with CSDS, and that there was a positive correlation between them, not affected by adjustment for related covariates.

We hope that large-sample, long-term prospective cohort studies could be conducted to confirm this relationship and investigate the possible underlying mechanisms, which may ultimately translate into better mental and physical health in older age.

## Data Availability

No datasets were generated or analysed during the current study.
